# Dispatching plasma membrane cholesterol and Sonic Hedgehog dispatch: two sides of the same coin?

**DOI:** 10.1042/BST20210918

**Published:** 2021-09-13

**Authors:** Kristina Ehring, Kay Grobe

**Affiliations:** Institute of Physiological Chemistry and Pathobiochemistry, University of Münster, Münster, Germany

**Keywords:** cholesterol, dispatched, Hedgehog, patched, shedding, sterol sensing domain

## Abstract

Vertebrate and invertebrate Hedgehog (Hh) morphogens signal over short and long distances to direct cell fate decisions during development and to maintain tissue homeostasis after birth. One of the most important questions in Hh biology is how such Hh signaling to distant target cells is achieved, because all Hh proteins are secreted as dually lipidated proteins that firmly tether to the outer plasma membrane leaflet of their producing cells. There, Hhs multimerize into light microscopically visible storage platforms that recruit factors required for their regulated release. One such recruited release factor is the soluble glycoprotein Scube2 (Signal sequence, cubulin domain, epidermal-growth-factor-like protein 2), and maximal Scube2 function requires concomitant activity of the resistance-nodulation-division (RND) transporter Dispatched (Disp) at the plasma membrane of Hh-producing cells. Although recently published cryo-electron microscopy-derived structures suggest possible direct modes of Scube2/Disp-regulated Hh release, the mechanism of Disp-mediated Hh deployment is still not fully understood. In this review, we discuss suggested direct modes of Disp-dependent Hh deployment and relate them to the structural similarities between Disp and the related RND transporters Patched (Ptc) and Niemann-Pick type C protein 1. We then discuss open questions and perspectives that derive from these structural similarities, with particular focus on new findings that suggest shared small molecule transporter functions of Disp to deplete the plasma membrane of cholesterol and to modulate Hh release in an indirect manner.

## Introduction

Hedgehog (Hh) morphogens control growth and patterning during development. The Hh spreading mechanism is especially intriguing because all Hhs are synthesized as C-terminally cholesterylated and N-terminally palmitoylated molecules. Both lipidations tether the 20 kDa Hh molecules to the membrane of producing cells. There, Hhs associate with lipid rafts, cholesterol-enriched membrane microdomains that serve as local organizers for the assembly and trafficking of multiple signaling molecules and their receptors [1,2], and assemble into large signaling platforms that range in size from 200 kDa to 1 MDa [3]. In *Drosophila* and in mammalian systems, Hhs are then solubilized from these platforms and travel to target cells at a significant distance from the source (∼300 µm in vertebrates and ∼50 µm in the fly) in order to bind to their receptor Patched (Ptc, also known as Ptch1). Possible mechanisms for Hh release and relay include Hh transport with lipoprotein particles [4], long-range transport via thin cellular extensions called cytonemes [5] and association of the vertebrate Hh family member Sonic Hh (Shh) with the soluble glycoprotein Scube2 (Signal sequence, cubulin (CUB) domain, epidermal-growth-factor (EGF)-like protein 2) [6–8]. Another possible means of releasing biologically active Shh from the plasma membrane is proteolytic Hh morphogen processing from its lipidated peptide termini — a process called shedding — as demonstrated *in vitro* [9,10] and *in vivo* [11,12]. In contrast with Scube2-assisted Shh transport, the latter model postulates that Scube2 merely serves as a Hh shedding enhancer rather than as a chaperone for dual-lipidated Shh [13,14].

For maximal activity, Scube2 requires expression of the twelve-pass transmembrane (TM) protein Dispatched1 (Disp) at the surface of Hh-producing cells [15–19]. In the absence of Disp expression, lipidated Hhs are retained in mouse and *Drosophila melanogaster* tissues that would otherwise secrete them [15,18], and long-range morphogen gradients do not form. Although the exact mode of Disp function is unknown, its activity is highly conserved across species because targeted expression of murine Disp rescues developmental defects associated with *Drosophila* Disp mutants [18]. Despite extensive research over the past decades, the precise molecular mechanisms controlling Disp-facilitated Hh release from the cell membrane remained unclear. This is about to change because of the power of cryo-electron microscopy (cryo-EM) to analyze proteins with or without their interactors being close to their native state: In the past three years, several cryo-EM-resolved structures of Disp, as well as related Ptc receptors, have provided new concepts about how these TM proteins may regulate Hh signaling [20–29]. These concepts were most recently complemented by biochemical studies on Disp biofunction that are in full agreement with its molecular structure [30].

## Disp displays the folding pattern of the RND superfamily of efflux pumps

**Figure 1.**
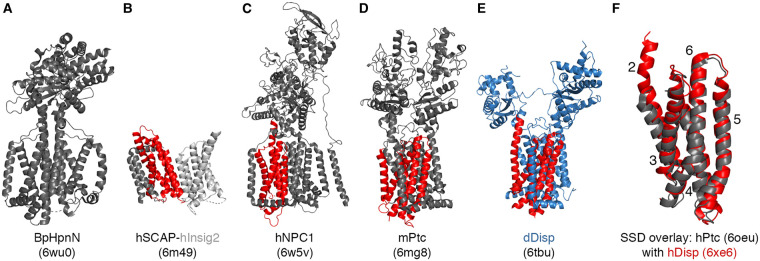
Similar conformations of eukaryotic TM and SSD domains. Shown are structures of prokaryotic RND transporter BpHpnN (**A**, pdb: 6wuO [39]) and human SCAP in complex with insulin-induced gene protein 2 (Insig2, **B**, pdb: 6m49 [37]), human NPC1 (**C**, pdb: 6w5v [40]), murine Ptc (**D**, pdb: 6mg8 [28]), and fly Disp (**E**, pdb: 6tbu [20]). SSDs (TM 2-6) are shown in red. **F**: Despite their two different extracellular loops, the TM SSDs of Ptc and Disp are very well aligned.

Several independent studies have revealed that *Drosophila* and human Disp possess a stereotypical RND TM topology [20,21] that resembles that found in bacteria, but most closely matches the TM topology of vertebrate Niemann-Pick type C protein 1 (NPC1) and the Hh receptor Ptc (Figure 1). A characteristic of all RND proteins is that their twelve TM domains are arranged into two pseudo-symmetrical parts, each consisting of six TM segments and one large extracellular globular domain [31]. This structure suggests that modern RND transporters may have originated from a duplication of ancestral RND progenitors comprising six TM helices with one loop inserted between the first and second TM domains. In functional terms, RND transporter superfamily members in Gram-negative bacteria are well-known drug-efflux pumps [31] that expel metal ions, low-molecular-weight antibiotics, oligosaccharides, sterols, and lipids [32,33]. Such multidrug recognition of substrates is based on their ‘loose' recognition as a result of permutations at numerous binding sites. Subsequently, these low-molecular-weight substrates are expelled through sequential conformational changes of the tripartite RND exporter and associated peristaltic motion of the substrate translocation channel. This process is powered by proton translocation across the bacterial membrane.

## Disp contains a highly conserved sterol-sensing domain

Although the eukaryotic RND proteins Ptc and Disp are also powered by distinct ion gradients across the plasma membrane (Disp uses Na^+^ influx and Ptc uses K^+^ efflux [34]), they seem to be more restricted in their substrate range, as suggested by their molecular structure. All vertebrate and invertebrate Ptc and Disp family members contain a sterol-sensing domain (SSD) that is conserved in proteins that bind, transport, or respond to cellular sterols, such as SREBP cleavage activating protein (SCAP) and NPC1 [35–37] (Figure 1). Consistent with such a putative function, sterol-like densities are present in the cryo-EM structures of Disp [20,21] and of Ptc [22,28], and Ptc can indeed transport cholesterol out of the membrane to indirectly maintain quiescence of the Hh pathway [28,38]. Hh binding to Ptc is thought to result in a structural rearrangement that alters the shape of the putative conduit to block cholesterol transport and thereby induce signaling. Consistent with this possibility, nanobody-mediated stabilization of an alternative conformation of Ptc is sufficient to induce potential allostery between the extracellular domain and the conduit to potently activate the Hh pathway *in vitro* and *in vivo*[29]. Such nanobody-associated functional arrest of Ptc cycling suggests that Ptc-modulated plasma membrane cholesterol can act as a second messenger in Hh pathway regulation [22,28]. Because the conserved SSD of Disp is virtually identical with that of Ptc (Figure 1), Disp can also be expected to export cholesterol, in this case to regulate Hh solubilization from the plasma membrane of producing cells. Such a cholesterol transporter function of human Disp has recently been demonstrated [30].

## Disp depletes cells of plasma membrane cholesterol

As mentioned earlier, the SSDs of Ptc and Disp can be superimposed on each other (Figure 1), as well as on the SSD of NPC1 [20], and all three molecules accommodate sterol-like densities in their hydrophobic conduits [20,22,28]. Together with the known capacity of Ptc to export sterol derivatives [28,38], Disp therefore emerged as another candidate to export free (unesterified) cellular cholesterol, 60%–80% of which resides in the plasma membrane [41]. A recent study supported this possibility [30] by demonstrating significantly reduced capacities of Disp knockout cells (Disp^−/−^) generated by CRISPR–Cas9 to export tritiated [^3^H]-cholesterol into the supernatant. Consistent with this finding, total cholesterol amounts in Disp^−/−^ cells were significantly increased and were restored to control levels (or to levels below that) by the cholesterol-extracting drug methyl-β-cyclodextrin (MβCD) and by the expression of transgenic Disp and Ptc^ΔL2^ (a Ptc variant lacking the second extracellular loop, making it insensitive to down-regulation of activity by Shh [42]) in Disp^−/−^ cells. These findings suggest that Ptc and Disp deplete free cholesterol from the plasma membrane, in full agreement with structural similarities between their SSDs (Figure 1) and the established role of SSDs to bind, transport, or respond to cellular sterols [36]. Such RND-transporter-like sterol efflux, however, requires a sink — possibly a soluble carrier — to transport the cholesterol away from the cell. In invertebrates, known cholesterol sinks are soluble lipoproteins called lipophorins. In vertebrates, high-density lipoprotein (HDL) represents a specialized lipoprotein fraction known to acquire peripheral cholesterol from ABC family transporters [43]. Indeed, the recent study also showed that serum lipoproteins increased [^3^H]-cholesterol egress, suggesting that extracellular cholesterol carriers such as HDL may act downstream of Disp (and possibly Ptc) to accept membrane sterols. The previous unrelated finding that Ptc can act as a lipoprotein receptor *in vivo* supports this possibility [44].

## Plasma membrane cholesterol amount or distribution modulates Scube2-dependent Shh shedding at the cell surface

Disp was discovered in a screen for segment polarity genes in *Drosophila* and Shh signaling modulators in mice [15,18], but not in a screen for defects in cholesterol homeostasis. This raises the question of how membrane cholesterol levels and the established role of Disp in regulated Shh release from the cell surface can be linked. One possible answer comes from the finding that cellular cholesterol depletion triggers shedding of several cell surface proteins *in vitro* [45–49] by the conversion of cellular precursors into their truncated soluble forms. Consistent with these reports, SDS–PAGE/immunoblotting revealed that Scube2 increased the solubilization of truncated Shh from Disp-expressing control cells and that it was exactly this truncated Shh fraction that Disp^−/−^ cells did not release [30]. Reverse-phase high-performance liquid chromatography (RP)-HPLC confirmed cholesteroylated Shh conversion into delipidated soluble proteins during Disp- and Scube2-enhanced release from control cells. This suggests Shh delipidation during Disp-dependent Shh shedding in an indirect, membrane-cholesterol-dependent manner. This idea was further supported by the findings that MβCD and transgenic Disp restored Shh shedding from Disp^−/−^ cells and that purified HDL increased Shh shedding from control cells but much less so from Disp^−/−^ cells. Notably, Shh shedding was also restored when Disp^−/−^ cells expressed the transgenic cholesterol transporter Ptc. This indicates that, like Ptc [28,38] and MβCD, Disp may deplete the plasma membrane of free (unesterified) cholesterol. Decreased plasma membrane cholesterol content or its changed distribution may then affect Shh shedding from the cell surface, at least under the serum-depleted conditions used in the study [30]. This new concept is supported by the structural conservation between Disp and Ptc and suggests a shared membrane-cholesterol export mechanism that may be essential not only for Hh perception in target cells [28], but also for Hh release from the plasma membrane of Hh-producing cells.

## The different role of Disp in the model of Scube2-mediated lipidated Shh release and transport

**Figure 2.**
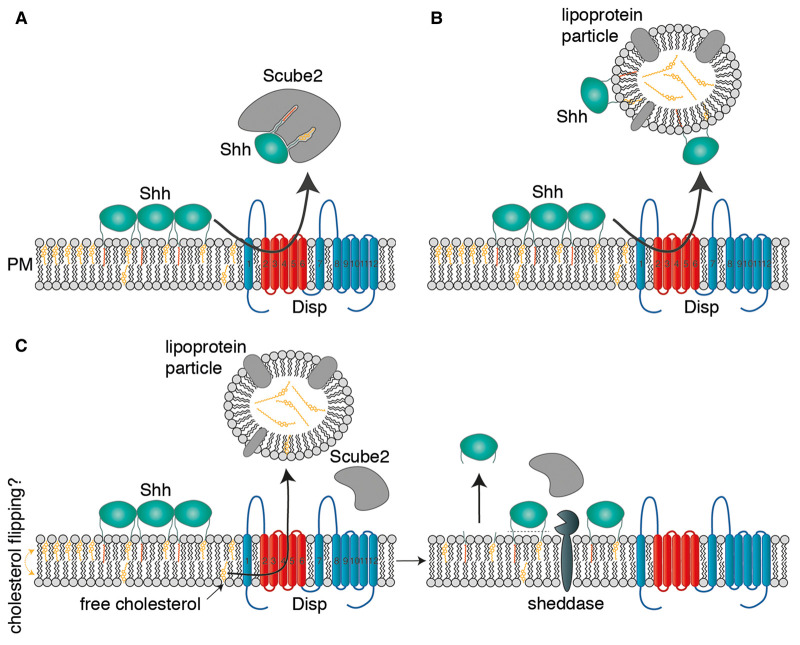
Models of Disp-mediated Shh release from the plasma membrane of producing cells. (**A**) Disp-mediated Shh extraction and handover to Scube2. (**B**) Lipidated Hh/Shh relay from the plasma membrane to soluble lipoprotein acceptors. (**C**) Disp-mediated cholesterol export to lipoprotein acceptors (left) depletes the plasma membrane of free cholesterol and is strongly associated with Scube2-assisted proteolytic Shh release, possibly by depleting lipid rafts of cholesterol and in turn allowing sheddase access to the substrate (right). This model integrates the established roles of Disp, Scube2, and lipoproteins in regulated Shh release and signaling.

Disp belongs to the RND family of small molecule transporters and contains TM-SSDs shared by the cholesterol pumps NPC1 and Ptc. Therefore, the experimental finding that Disp can expel membrane cholesterol is not surprising. The important question arising from this finding, however, is whether or how it correlates with current concepts of Hh solubilization. The model that Disp extracts dual-lipidated Shh proteins from the cell membrane to hand them over to Scube2, which in turn chaperones the insoluble morphogen to the Ptc receptor, was among the first, and therefore remained one of the most widely accepted, hypotheses (Figure 2A) [7,21,34]. Although the newly observed cholesterol exporter function of Disp does not explicitly preclude this possibility, it is also difficult to determine whether it can provide direct support for it. This is in part because published *in vitro* support for this model is not as clear as is generally assumed. For example, immunoblotted soluble Shh has been consistently considered to be dual lipidated [6–8,34]. Yet, immunoblotted soluble proteins cannot provide this information in the absence of side-by-side comparisons with dual-lipidated control proteins (the cellular precursor fraction) on the same blot; RP-HPLC of the entire spectrum of cellular and solubilized proteins, or autoradiography of [^3^H]-labeled terminal lipids would also be required to support the model. Such analyses, however, have not been presented in the studies. More recently, internal luciferase tags were introduced to track Shh solubilization [34] but, unlike terminal tags that consistently get removed in the process of Scube2-mediated Shh solubilization [14], internal tags cannot provide information on the molecular release mode. The model of Disp-mediated Hh handover to Scube2 is also difficult to align with the available *in vivo* data because overexpressed mouse Disp can replace *Drosophila* Disp function despite the fact that flies do not express Scube2 orthologs [18] and because vertebrates made deficient in Scube2 function show only mild developmental defects [50,51]. These observations suggest that the Disp-mediated Shh extraction and relay of the lipidated morphogen to Scube2 does not likely represent the only possible or main mode of Hh release. In addition to lacking clear functional links to Disp as a cholesterol exporter [30], the model of direct Hh extraction by Disp and handover to Scube2 can also not be easily linked to the established important role of lipoproteins in Hh solubilization or the presence of de-steroylated bioactive Hh and Shh variants *in vitro* and *in vivo* [4,52].

## Disp-controlled membrane cholesterol depletion can be linked with Shh shedding and established lipoprotein functions in Hh release

As described earlier, cellular cholesterol depletion triggers cell surface shedding of numerous proteins *in vitro* [45–49], providing a blueprint for Disp-mediated and cholesterol-dependent Shh shedding from the plasma membrane [13,14] (Figure 2C). Hh/Shh processing and loss of terminal lipids (including terminally inserted hemagglutinin tags) was previously confirmed by controlled immunoblotting, RP-HPLC, and autoradiography *in vitro* [9,13,14,53] and by functional inactivity of sheddase-resistant Hh variants *in vivo* [11,12,52]. Facilitated Shh shedding by Scube2 can be explained in two ways: First, the Scube2 CUB domain is required to increase Shh processing under serum-depleted conditions in a similar manner to that of the CUB domains of the extracellular procollagen C-proteinase enhancers PCPE1 and PCPE2 [13,14,54,55]. Second, several Scube2 EGF domains show high levels of amino acid identity with endocytic receptors of the low-density lipoprotein (LDL) receptor class (LDLR and LRP5) [56], and Scube2 EGF domains also show high levels of amino acid identity with some EGF domains of CUB, a molecule that can strongly bind HDL and apolipoprotein AI, which both accept and transport cholesterol [57,58]. These observations support the exciting option that Scube2 may be part of a complex with Disp that binds and recruits soluble acceptors of cholesterol, such as those resembling HDL. The demonstrated effect of exogenous lipoproteins as stimulators of Hh shedding [30] may explain the important role of these cholesterol acceptors for Hh biofunction and is fully in line with the observations that lipophorins enhance Hh long-range signaling [4] and that Ptc binds fly lipoproteins *in vivo* [44]. The newly postulated Disp mechanism of membrane cholesterol egress and modulated Hh shedding — two sides of one coin — is supported further by the detection of signaling of active, de-steroylated Hh/Shh forms *in vitro* and *in vivo* [52].

## Disp-controlled Shh shedding can be linked to models of non-diffusive Hh transport

One problem of Disp-controlled shedding is that it implies subsequent Shh diffusion to distant target cells. This, however, is difficult to envision for two main reasons: First, diffusion is too slow for Shh transport over long distances, and second, diffusion away from the cell surface would prevent most Shh to find its receptor Ptc and therefore diminish signaling [59]. Consistent with these considerations, a set of elegant cell-based gradient reconstitution assays showed that direct cell-cell contacts or a continuous extracellular matrix are required for Shh movement *in vitro*, because Shh gradient formation and signaling within cell layers was unaffected by media flow but was completely blocked by small gaps between Shh producing and receiving cells [60]. The latter finding bears striking resemblance to the long-standing observation that Hh cannot cross even a small gap lacking proper expression of the extracellular matrix constituent heparan sulfate (HS) *in vivo* [61]. HS consists of negatively charged sugar chains at the cell surface with different degrees of sulfation [62]. HS-dependent Hh transport was previously explained by unspecific electrostatic binding of Hh/lipophorin complexes to HS as a prerequisite for subsequent Hh binding to Ptc [63] or — rather indirectly — by HS-dependent guidance and growth of long, specialized cytonemes that connect Hh-producing and receiving cells *in vitro* [5] and *in vivo* [64–66]. Of note, Shh co-localizes with Disp in cytonemes [5], and Hh reception takes place in membrane contact sites between Hh-sending cytonemes and Ptc-receptors on Hh-receiving cytonemes [65]. These findings suggest that Disp-controlled shedding may act at these sites to combine cytoneme-mediated Shh long-range transport with short-range Shh relay to target cells. Alternatively, sheddases may ‘unpack' Hh from soluble exosomes after their release from cytoneme tips as a prerequisite for unimpaired Hh binding to Ptc. Note that, in both cases, Disp would act locally — and therefore effectively — on cellular membranes that cannot be easily replenished with free cholesterol. In this regard, Disp would again resemble Ptc that locally decreases the pool of free cholesterol at the membrane of primary cilia to effectively inhibit signaling [67].

## Perspectives

- *Importance:* New findings support that Disp and Ptc, likely via their SSDs, remove cholesterol from the plasma membrane and that soluble lipoproteins can accept Disp-expelled cholesterol (Figure 2). Regulated plasma membrane cholesterol amounts or plasma membrane-microdomain distribution may therefore not only act as Ptc-controlled second messengers in Hh signaling at receiving cells, as shown previously, but may also play an unanticipated role in facilitating Shh release from the plasma membrane of producing cells *in vitro*.
- *Current thinking:* Although structural analyses revealed different possible modes of Hh binding to Ptc and subsequent signaling, the current thinking is that Disp extracts dual-lipidated Shh to hand it over to Scube2. Although cryo-EM of Ptc together with its dual-lipidated Shh ligand provided conceptual support for the model, it is important to note that the membrane-extracted, dual-lipidated Shh used to generate these structures clearly corresponds to the Shh pre-release state upstream of Disp and Scube2 function. It is less clear and remains to be demonstrated whether Shh deployed by Disp and Scube2 always retains both lipids.
- *Future directions:* The future of Disp-research may elucidate unexplored roles of Scube2 in Disp-mediated sterol transport. Another important direction is to analyze possible links between Disp-regulated shedding and other modes of Hh gradient formation, such as cytoneme-mediated Hh transport. In addition, from the loose substrate binding of bacterial multidrug efflux pumps, cholesterol export may not be the only function of Disp: It remains conceivable that the cholesteroylated Hh/Shh C-terminus may also be recognized and expelled by Disp, which would reveal another two-sided Disp function. Such an additional Disp function would link seemingly disparate models of Hh release and transport by defining their preferred requirements for generating lipidated and delipidated Hh ligands with different activities and signaling ranges from one plasma-membrane-associated precursor.

## Abbreviations

cryo-EM: cryo-electron microscopy
CUB: cubulin
Disp: Dispatched1
EGF: epidermal growth factor
HDL: high-density lipoprotein
Hh: Hedgehog
LDL: low-density lipoprotein
MβCD: methyl-β-cyclodextrin
NPC1: Niemann-Pick type C protein 1
Ptc: Patched1
RND: resistance-nodulation-division
RP-HPLC: reverse-phase high-performance liquid chromatography
Scube2: Signal sequence, Cubulin domain, EGF-like protein 2
Shh: Sonic hedgehog
SSD: sterol-sensing domain
TM: transmembrane
